# Mitochondria-based holistic 3PM approach as the ‘game-changer’ for individualised rehabilitation—the proof-of-principle model by treated breast cancer survivors

**DOI:** 10.1007/s13167-024-00386-0

**Published:** 2024-11-20

**Authors:** Martin Pesta, Barbara Mrazova, Marko Kapalla, Vlastimil Kulda, Eleni Gkika, Olga Golubnitschaja

**Affiliations:** 1 Department of Biology, Faculty of Medicine in Pilsen, Charles University, Plzen, Czech Republic; 2 F. D, Roosevelt University Hospital, Banska Bystrica, Slovakia; 3 Department of Medical Chemistry and Biochemistry, Faculty of Medicine in Pilsen, Charles University, Plzen, Czech Republic; 4 Department of Radiation Oncology, University Hospital Bonn, Rheinische Friedrich-Wilhelms-Universität Bonn, 53127 Bonn, Germany; 5 Predictive, Preventive and Personalised (3P) Medicine, Department of Radiation Oncology, University Hospital Bonn, Rheinische Friedrich-Wilhelms-Universität Bonn, 53127 Bonn, Germany

**Keywords:** Predictive preventive personalised medicine (PPPM / 3PM), Breast cancer survivors, Secondary care, Life quality, Rehabilitation; Chronic fatigue, Patient-friendly non-invasive approach, Tear fluid analysis, Mitochondrial health, Homeostasis, Mitophagy, Health risk assessment, Sleep medicine, Behavioural patterns, Individualised patient profile, Patient self-report, Healthcare economy, Health policy, Expert recommendations

## Abstract

Breast cancer belongs to the most commonly diagnosed malignancies worldwide, with its increasing incidence paralleled by advances in early diagnostics and effective treatments resulting in significantly improved survival rates. However, breast cancer survivors often experience significantly reduced quality of life linked to the long-term health burden as a consequence of aggressive oncological treatments applied. Their most frequently recorded complains include chronic fatigue, reduced physical activity, disordered sleep, chronification of pain, and severe mental health impairments—all per evidence are associated with compromised mitochondrial health and impaired homeostasis. Self-report of a breast cancer survivor is included in this article to illustrate currently uncovered patient needs. This article highlights mechanisms behind the suboptimal health of breast cancer survivors associated with mitochondrial damage, and introduces a novel, mitochondria-based holistic approach addressing rehabilitation concepts for breast cancer survivors following advanced principles of predictive, preventive and personalised medicine (3PM). By operating via mitochondrial function, the proposed holistic approach triggers systemic effects at molecular, sub/cellular and organismal levels positively affecting energy metabolism, repair mechanisms as well as physical and mental health creating, therefore, highly effective rehabilitation algorithms tailored to an individualised patient profile. The proposed methodology integrates mitochondrial health assessments utilising mitochondrial homeostasis biomarkers in tear fluid as a non-invasive diagnostic tool, tailored nutraceuticals and lifestyle adjustments. The introduced approach aligns with advanced principles of 3PM, offering a holistic and proactive framework for managing persistent post-treatment symptoms of suboptimal health in the cohort of cancer survivors. Furthermore, presented approach is also applicable to pre-habilitation programmes considering needs of other patient cohorts affected by chronic diseases such as CVD and orthopaedic disorders with planned major surgical incisions, who require individually adapted pre- and rehabilitation programmes. Implementing such innovative pre- and rehabilitation strategies may lead to a full recovery, sustainable health conditions and, therefore, facilitating patients’ comeback to normal daily activities, family and professional life. Contextually, presented approach is considered a ‘proof-of-principle’ model for the 3PM-related paradigm shift from reactive medicine to a cost-effective holistic health management in both primary and secondary care benefiting a large spectrum of affected patient cohorts, individuals in suboptimal health conditions as well as society at large.

## Preamble

**Fig. 1:**
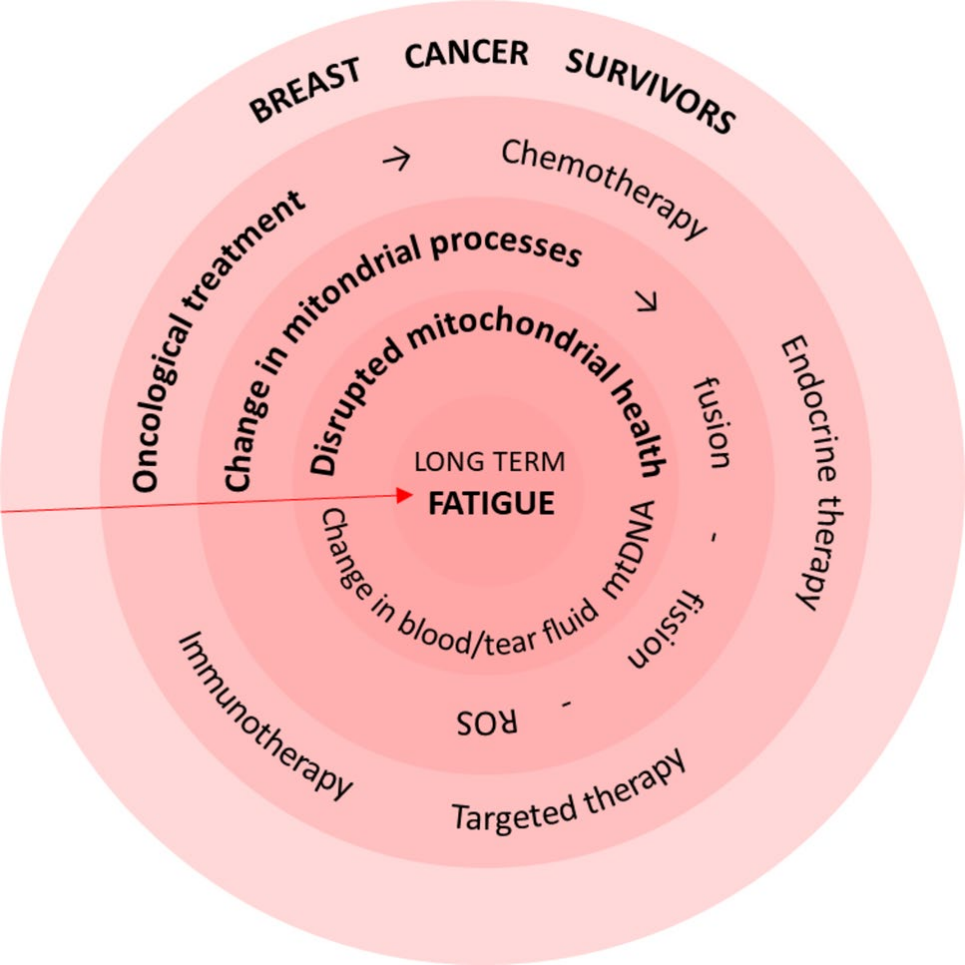
Breast cancer survivors as a prominent example for the paradigm change towards proactive post-treatment health management and innovative rehabilitation programmes

Breast cancer (BC) is the most frequently diagnosed cancer globally. Its incidence is rising steadily by 1.44% annually [1] and may, in part, be associated with the global increase in life expectancy [2]. Due to advancements in screening, early diagnosis, and timely treatments, the 10-year survival rate for breast cancer patients is increasing e.g. in Germany reaching currently 83% (as recorded in 2020) [3]. Oncological disease and routinely applied treatments (surgery, radiotherapy, chemotherapy, targeted therapy, immunotherapy, and hormonal therapy) influence all aspects of daily life. Besides the physical strain (and the associated fatigue) itself, patients face emotional stress and fear of recurrence for a long period after treatment. As a result of more successful treatment of BC, there is a growing number of BC-survivors facing unique long-term physical, psychological, and psychosocial changes, which can significantly impact their quality of life [4]. To this end, socioeconomic aspects are crucial, as, for example, only 57% of female BC-survivors in Germany return to the same job position [5]. All these issues summarised by Fig. 1 remain unsolved and attract a lot of attention of multi-professional groups focused on creating innovative rehabilitation programmes benefiting affected patient cohorts, family members, and society at large.

## Reduced quality of life after breast cancer (BC) treatments with associated socio-economic aspects

Both cancer and its treatment are associated with the most commonly reported challenges amongst women during the immediate post-treatment period (up to one year), including issues related to physical function, emotional well-being, and treatment-related side effects. In the longer term (one to four years post-treatment), persistent fatigue becomes a predominant concern. These health-related consequences significantly influence the ability to resume pre-diagnosis lifestyles, including the capacity to return to employment [6].

Up to 93% of breast cancer survivors experienced on-therapy side effects or subsequently long-term side effects after the treatment. These symptoms may involve physical, functional, emotional, and psychosocial changes, which can dramatically alter their quality of life [7]. Worse quality of life after breast cancer treatment meets the characteristics of suboptimal health, particularly affecting physical, emotional, and social functioning [8–10].

### Returning to work after breast cancer

Return to the work (RTW), whether to their original or a different position, is related to their overall health conditions, including both physical and mental health. For breast cancer survivors, RTW is also crucial from economic, societal, and personal perspectives. One year after surgery, 57% of survivors returned to their previous work, while 22% worked reduced hours compared to their pre-diagnosis employment. Significant associations were found between impaired RTW after 1 year and factors such as depressive symptoms, arm morbidity, lower education levels, and younger age. Additionally, persistent physical fatigue and living with a partner were linked to impaired RTW after 5 years. Major self-reported reasons for difficulties in returning to work included fatigue and cognitive issues [5, 11, 12].

### Side effects of breast cancer treatments

Despite recent advancements, modern oncological treatment of BC continues to be associated with a wide range of side effects, varying in severity. Furthermore, due to the ineligibility of some patients for targeted therapies, many are still treated with cytostatic and cytotoxic chemotherapy. While acute side effects, both during treatment and shortly after, are typically managed in the course of oncological care, long-term side effects often pose significant challenges, particularly for breast cancer survivors. These long-term side effects can substantially impact quality of life and are shaped by the specific treatment regimen employed, as well as generalised issues observed across the majority of treated patients. The manifestation of these systemic therapy-related side effects is highly individualised, influenced by factors such as patient age, menopausal status, and the presence of comorbidities, including hypertension [13, 14].

### Chemotherapy side effects

Chemotherapy is associated with several severe side effects, some of which can be life-threatening both during and after treatment. Amongst these, chemotherapy-induced nausea and vomiting (CINV) is a significant concern. CINV is typically classified into five distinct categories: acute, delayed, anticipatory, breakthrough, and refractory [15]. The most common long-term side effects of chemotherapy include fatigue, insomnia, peripheral neuropathy, cognitive impairment, oestrogen deprivation, cardiotoxicity, and the development of secondary malignancies. These chronic toxicities can affect a substantial proportion of breast cancer survivors, profoundly impacting their quality of life. Although guidelines exist for managing specific adverse effects, such as peripheral neuropathy, comprehensive approaches for addressing the full spectrum of long-term chemotherapy-induced toxicities remain limited [16].

### Immunotherapy agents

Enhancing the immune response through immune checkpoint inhibitors (ICIs) carries the risk of developing immune-related adverse events (irAEs). These irAEs are autoimmune conditions that can impact any organ system and are distinct from the adverse effects typically associated with chemotherapy [17]. Toxicity in breast cancer patients treated with immune checkpoint inhibitor monoclonal antibodies is primarily associated with T-cell hyperactivity [18].

The manifestations of immune-related adverse events (irAEs) are highly diverse. The most commonly observed irAEs, particularly in treatments combining immune checkpoint inhibitors with chemotherapy, include arthralgia, thyroid dysfunction (both hypothyroidism and hyperthyroidism), transaminitis, dermatologic toxicities, pneumonitis, and colitis. Fatigue is also frequently reported, though generally mild, with an estimated occurrence rate of 16 to 24%. Combining immunotherapy with chemotherapy or dual immunotherapy is linked to a higher incidence of irAEs compared to using ICIs alone [17].

Prior to initiating treatment, a thorough assessment of each patient’s susceptibility to developing these adverse events is essential [19]. A personalised, risk-based preventive approach, incorporating tailored immunosuppressive strategies, has been proposed as one of the most promising future directions in managing irAEs [18, 20].

### Endocrine therapy

Endocrine therapies may be recommended for up to 10 years in women with early stage disease after surgery [21]. While some patients report few or manageable side effects from endocrine therapy, others experience severe and prolonged symptoms [22]. Due to the extended duration of endocrine therapy, patients often face both on-treatment side effects—such as hot flashes, fatigue, muscle pain, mood changes, vaginal symptoms, gastrointestinal issues, headaches, weight gain, and bone density loss—as well as long-term effects, including an increased risk of heart disease and stroke, osteoporosis, memory problems or difficulty with concentration, secondary cancers, sexual dysfunction, chronic fatigue, joint pain and stiffness, and metabolic changes [23, 24]. More than a third of breast cancer survivors taking endocrine therapy report hair thinning and loss, described as frontal and parietal hairline recession [25]. Side effects of endocrine therapy are particularly predominant amongst premenopausal patients taking GNRH agonists for ovarian suppression [26].

### Targeted therapy

The routine use of targeted therapies, whose mechanisms of action differ from the conventional cytotoxic agents, has also led to the emergence of new toxicities, such as metabolic disorders [27]. Although many targeted therapies have fewer listed side effects compared to traditional chemotherapy, their acute toxicities can become subacute and more prolonged due to the extended duration of treatment (e.g. 2 years for abemaciclib in the adjuvant setting). Some toxicities, such as interstitial lung disease from abemaciclib, can be fatal, though rarely. The long-term toxicities in the adjuvant setting have yet to be fully characterised [28, 29].

### Illustration by the self-report of a BC-survivor

A 33-year-old female patient made a successful academic carrier as a medical doctor. She is married and gave birth to two healthy children. BMI = 14.87 (weight 40 kg, height 164 cm).

Family history: CVDs and diabetes mellitus type 2. However, nobody in her family was diagnosed with any genetic or cancer diseases.

#### Breast cancer diagnosis history

In the 16th week of her second pregnancy, she discovered a lump on her breast. At that time, she was still breastfeeding her first 20-month-old son. At approximately 17 weeks of the pregnancy, she underwent a breast ultrasound and a month later biopsy. After examinations at 25 weeks of the pregnancy, the diagnosis was finalised: triple negative breast cancer with metastasis in the node, invasive intraductal carcinoma with negative genetics. The complication was that the tumour was connected to a cyst. It quickly filled and grew, created oppression, and must be repeatedly evacuated. She underwent 3 chemotherapy treatments (FEC) during pregnancy. The tumour has shrunk only slightly, but at least the cyst has shrunk significantly. Seven weeks later, she gave birth to a healthy daughter who was 3700 g heavy and 52 cm long. It was natural spontaneous birth, 5 days after the due date. At the time of delivery, the tumour had grown significantly. Subsequently, she underwent 3 chemotherapy treatments (docetaxel). During the oncological treatment, the cyst quickly filled up again and had to be drained every 2 weeks, in the last week even every 3–4 days. The tumour was painful; analgesics had no effect. Due to repeated injections in the tumour site, the skin gradually thinned until necrosis occurred. Chemotherapy was not effective, so it was terminated after 3 cycles (out of the planned 4). In the further course, a radical mastectomy was performed and 10 nodes were removed from the axilla. The operation ended with no particular complications despite a remaining large stretched scar.

#### Complications recorded after the BC-treatment

Her mental and emotional health suffered a lot due to the diagnosis. She listened to podcasts, read specialised books, went to psychologists, but almost none was of help. She was trying to understand the origin of the disease development. It took her almost a year before she realised that her health burden may be caused by psycho-traumas happened mainly in her childhood. She changed her psychologist and the meetings started to help. Her relationship with family members turned to more positive. The strength was always her warm relationship with friends that makes her happy. She feels that motherhood is what she was created for, despite the persistent burn out due to much work towards entire household and both children, being on parental leave for altogether 4.5 years till now. All family members (both parents and their children) are extremely stress-sensitive. Two years after the end of the BC-treatment, she is still physically completely exhausted and suffers a lot from chronic fatigue.

She noticed that after she underwent chemotherapy, about 3-times a year her eyelashes fall out, and it takes a month till they reach a full length again. Her hair texture has changed.

Before the BC treatment, she never suffered from any skin problems, besides skin dryness which persists. A year after the treatment, she developed rosacea and got pityriasis versicolor 1.5 year after the treatment.

Her gastrointestinal problems persist: she was lactose and casein intolerant and became additionally eggs intolerant after BC-treatment.

Her worries and anxiety were strongly pronounced after BC diagnosis and treatments. However, stepwise she starts to believe to be cured. She is physically very active life-long.

Dietary patterns: fish, meat (turkey, chicken, sometimes beef), and daily, all kinds of vegetables and fruits. Excluded products are eggs, sugar (only natural in small quantities), smoked products, alcohol, pork, semi-ready products, sweets, and foods of poor quality content.

Drinking regime: she drinks mostly pure water, unsweetened herbal and green teas—at least 1.5 l daily.

Her sleep is of poor quality: very rarely, she sleeps through the whole night, usually not more than 4 h and rarely 7 h; children wake up often, up to 5 times a night. Sometimes, she has troubles to fall asleep despite tiredness. She goes to bed together with her children, wakes up with them, and sleeps with them during the day.

#### Phenotyping

She demonstrates characteristic symptoms and features of the Flammer Syndrome Phenotype (FSP). She always feels very cold and considers comfortable room temperatures around 30 °C. Specialised survey [30] revealed following characteristic symptoms and signs of the FSP:

- Low BMI (yes, extremely pronounced)
- Stress sensitivity (yes, extremely pronounced)
- Frequently cold extremities (yes, extremely pronounced)
- Low blood pressure (yes)
- Dizzy (yes)
- Pain perception (strong)
- Prolonged time to fall asleep and shifted circadian rhythms (yes)
- Perfectionism/meticulous personality (yes, extremely pronounced).

Further, during both entire pregnancies, pronounced nausea and vomiting have been recorded for the patient that may reflect an increased sensitivity to metabolic and cardio-vascular stress typical for the FSP carriers [31].

Flammer syndrome is a condition characterised by an abnormal vascular response to various stimuli such as cold exposure and any kind of stress provocation. FSP carriers commonly experience phenotype-characteristic symptoms and signs listed above. Flammer syndrome is more prevalent amongst pre-menopausal females being detectable early in life starting with puberty that makes FSP-related screening and medical coaching very reasonable in young populations following principle of 3PM in cost-effective healthcare [31].

Further, mounting research evidence demonstrates a predisposition of FSP carriers to aggressive BC disease course on one hand and one the other hand, compromised mitochondrial health which is generally characteristic for the FSP [32–35]. Contextually, mitochondria-based approach is strongly recommended for advanced diagnostics and treatments applied to the FSP carriers in primary (protection against health-to-disease transition) and secondary (protection against disease progression) care [31, 36–38]. To this end, tear fluid is considered an optimal source of information for predictive diagnostics applied to the FSP carriers early in life starting with puberty [38, 39].

## All around mitochondrial health and homeostasis under BC treatments

The subjective experience of fatigue, a common symptom during the post-treatment phase of various oncological therapies, can be linked at the cellular level to the functioning of the energy metabolism, with mitochondria being the central players in this process. This concept is reflected in the term ‘mitochondrial health’ [40].

Mitochondrial health and homeostasis refer to the state of optimal functioning and balance within the mitochondria and are with direct relation to a quantity of effective mitochondria. Over 90% of ATP, as the main source of energy for cellular processes, is produced in the mitochondria through oxidative phosphorylation, the Krebs cycle, and β-oxidation of fatty acids. Therefore, mitochondrial health and homeostasis crucially influence the overall functioning and performance of the organism [41].

Although the structural organisation of mitochondria is identical in all cells of the human body, a significant difference in the quantity of mitochondrial processes can be observed, particularly in cells rich in mitochondria, such as those in energy-demanding tissues. This implies that deviations from mitochondrial homeostasis manifest differently and have varying consequences across cell types. Mitochondria in skeletal muscle and cardiac muscle cells are elongated and larger compared to mitochondria in other tissues. Their inner membrane contains more folds (cristae), which increases the surface area available for ATP production, reflecting their high energy production demands [42]. In contrast, mitochondria in liver cells have fewer cristae than muscle cells, as their energy requirements are not as extreme. However, their metabolic functions remain extensive. Each cell of liver tissue contains 1–2 thousand mitochondria, occupying about 20% of the hepatocyte volume [43].

The quantity of effective mitochondria is assured by processes mitochondrial biogenesis, mitochondrial dynamics, and mitophagy. These processes also have a key influence on the proper functioning of mitochondria as organelles and on the adaptation to the cell energy needs [44].

### Mitochondrial biogenesis

Mitochondria do not arise de novo but through the division of existing mitochondria. Despite this autonomous replication, their function depends on the expression of genes encoded by the nuclear genome. Mitochondrial DNA encodes only 13 genes directly involved in mitochondrial function, specifically in ATP production. A key regulator of mitochondrial biogenesis is peroxisome proliferator-activated receptor gamma coactivator 1-alpha (PGC-1α), which activates nuclear transcription factors NRF1 and NRF2, along with mitochondrial transcription factor A (TFAM). These factors orchestrate biogenomic coordination between the nuclear and mitochondrial genomes by regulating the expression of nuclear-encoded proteins in the electron transport chain (ETC) [45].

Mitochondrial biogenesis is also modulated by signalling pathways, including AMP-activated protein kinase (AMPK) and sirtuin 1 (SIRT1), which respond to cellular energy stress and fluctuations in energy status [46]. Additionally, the expression of proteins responsible for transport complexes, such as translocase of the outer membrane (TOM) and translocase of the inner membrane (TIM), as well as transporters like ADP/ATP translocase and various metabolite carriers, plays a crucial role in mitochondrial function [47].

### Mitochondrial dynamics

The balanced fission and fusion processes, referred to as mitochondrial dynamics, coordinate mitochondrial shape, size, number, energy metabolism, cell cycle, mitophagy, and apoptosis. Mitochondrial dynamics at the same time allows mitochondria to respond to changing conditions both within the mitochondria and in the cytoplasm. The dynamics of mitochondria are regulated by proteins mitofusin (MFN1 and MFN2) and OPA1, which are involved in fusion, and dynamin-related protein 1 (DRP1) and FIS1, which regulate division. The balance between fusion and division is critical for maintaining of mitochondrial health [48]. An imbalance between these opposing events alters mitochondrial dynamics, affects the overall mitochondrial shape, and deregulates mitochondrial function [49].

### Mitophagy

Mitophagy removes damaged or dysfunctional mitochondria, preventing the accumulation of dysfunctional mitochondria that can lead to cellular damage and subsequently result in disease. Mitophagy is activated in response to stress conditions, including loss of mitochondrial membrane potential, oxidative stress, hypoxia, and nutrient deprivation, amongst others. Key regulators of mitophagy include the proteins PINK1 (PTEN-induced kinase 1) and Parkin. PINK1 accumulates on the surface of damaged mitochondria and recruits Parkin, which ubiquitinates mitochondrial proteins, leading to their degradation. Mitophagy is also regulated by the mTOR (mechanistic target of rapamycin) signalling pathway, which affects general autophagy and thereby mitophagy [50].

### Pharmacological cancer treatment and mitochondrial health

It is generally recognised that all oncological treatments affect several aspects of mitochondrial life cycle and function, with varying degrees of impact depending on the cell type. Breast cancer therapies involve multiple modalities, each exerting distinct effects on mitochondrial processes. Despite the variability of these effects, the impact of oncological interventions frequently manifests in compromised mitochondrial health, often experienced by patients as increased fatigue. This fatigue can be so pronounced, as previously noted, that it significantly impairs quality of life during the post-treatment period.

Pharmacological cancer treatments influence mitochondrial processes both directly and indirectly. The ratio of direct to indirect effects on mitochondrial populations, and consequently their function, varies across treatment modalities. Direct influence is seen, for instance, in targeted therapies that inhibit the mTOR pathway, thereby directly impacting mitophagy [51]. In contrast, indirect influence occurs with treatments like immunotherapy, where inflammation as a side effect affects mitochondrial function [52].

Chemotherapy, immunotherapy, targeted therapy, and endocrine therapy can all result in mitochondria-related side effects, including dysregulation of mitochondrial biogenesis, dynamics, and mitophagy- all ultimately leading to insufficient and even impaired ATP production, and therefore, insufficient energy supply at cellular and organismal levels. While most treatment-related side effects are managed during cancer therapy, chronic fatigue persists in a significant portion of treated patients. This persistent fatigue is directly linked to disfunction of the affected cellular energy apparatus, particularly to damaged mitochondria.

### How endocrine therapy affects mitochondrial health

Hormones such as oestrogens and androgens can directly influence mitochondrial functions. For example, evidence suggests that oestrogens have a protective effect on mitochondria, so their blockage (e.g., through aromatase inhibitors or antioestrogens) can disrupt this protective function [53]. The ability of oestrogen to increase manganese superoxide dismutase (MnSOD) levels, an enzyme crucial for maintaining cellular redox balance and preventing oxidative stress, is ablated in ERα KO mice but not in ERβ KO mice. These data show that ERs increase mitochondrial antioxidant production [54]. As a result, some patients undergoing endocrine therapy experience side effects such as fatigue, which can be caused by mitochondrial dysfunction [24].

### How chemotherapy affects mitochondrial health

Chemotherapy is still extensively used to treat BC. There is an accumulated evidence that this modality of treatment has substantial negative impact on mitochondrial health and affects several mitochondrial processes. Mitochondrial alterations subsequent to chemotherapy include a reduction in mitochondrial biogenesis, altered mitochondrial dynamics, mitophagy defects, increase of H_2_O_2_ production, and increased initiation of apoptosis. All of these alterations likely explain, at least in part, the high prevalence of skeletal muscle and cardiorespiratory deconditioning classically observed in BC patients [55].

Chemotherapeutics may also alter the dynamics of mitochondrial fission and fusion. On the molecular level, it was observed that DNA-damaging chemotherapeutics increased mitochondrial elongation and OXPHOS, but microtubule poisons (e.g. taxanes) increased mitochondrial fragmentation [56, 57]. Chemotherapy also targets various mitochondrial enzymes or proteins, including mitochondrial ribosome and ribosomal proteins (MRP), DNA polymerase subunit gamma (PolG), ATP synthase, and the calcium or other ion channels as the voltage-dependent anion channel 1 (VDAC1), the mitochondrial calcium uniporter (MCU) complex, and the mitochondrial permeability transition pore complex (mPTPC) [58].

### How immunotherapy affects mitochondrial health

Immunotherapy treatments like ICIs and CAR T-cell therapy have shown long-term results in patients with advanced cancer, but they can cause serious, even fatal, inflammatory, and immune-related side effects [52].

One of the reported side effects of immunotherapy with a significant impact on human metabolism is colon inflammation. The frequently early onset of colitis symptoms following treatment initiation is associated with a striking accumulation of CD8 T cells with highly cytotoxic and proliferative states and no evidence of regulatory T cell depletion. T cell receptor (TCR) sequence analysis demonstrated that a substantial fraction of colitis-associated CD8 T cells originated from tissue-resident populations [59]. Perturbed mitochondrial dynamics, particularly related to the dynamics of mitochondrial fission, may be a key feature of intestinal inflammation [60].

### How targeted therapy affects mitochondrial health

As already mentioned, the use of targeted therapy causes toxicities such as metabolic disorders [27]. Tyrosine kinase inhibitor (TKI), especially nilotinib, is associated with an increased risk of hyperglycaemia in patients with a history of diabetes mellitus and prediabetes at baseline and age over 60 years; furthermore, dyslipidaemia at baseline was identified as an additional risk factor for development of hyperglycaemia on imatinib treatment [61].

TKIs can also cause glycidic disorders (brigatinib, sorafenib, sunitinib, and vandetanib) and lipid metabolism alterations (ruloxitinib, idelasib, lenvatinib, and lorlatinib). Notably, both glycidic and lipid disorders have been described during nilotinib and ponatinib administration [62]. Disorders of glucose and lipid homeostasis are reported in a significant number of patients, especially with mTOR-PI3K targeting [63]. Such agents may lead to hyperglycaemia by interrupting the intracellular response to insulin, causing decreased glucose transport and glycogen synthesis, and increased glycolysis. In addition, chronic inhibition of mTOR has been linked to decreased proliferation and reduction of insulin producing pancreatic β-cells, contributing to both hyperglycaemia and the development of insulin resistance [64, 65]. At the molecular level, both alterations in lipid metabolism and disturbances in glucose metabolism meet in the citrate cycle running inside the mitochondria and thus adversely affect their function.

## Mitochondrial health and homeostasis as the ‘game-changer’ in the rehabilitation quality and individual outcomes

Chronic fatigue, linked to mitochondrial dysfunction, involves the inability to meet energy demands efficiently, manifesting as persistent exhaustion. Mental health issues, such as anxiety, depression, and panic attacks, can also be influenced by mitochondrial dysfunction. A significant link between mitochondrial dysfunction and mood disorders is established, highlighting the role of energy imbalance and oxidative stress in mental health. Chronic stress alters mitochondrial bioenergetics. This is especially evident in conditions like depression correlating with impaired cellular energy production.

Pain management utilising lasting application of painkillers is known to have significant negative side-effects towards systemic mitochondrial damage and impaired homeostasis [66]. Per evidence, under health conditions linked to the pain chronification, mitochondrial ATP production is significantly decreased, and imbalanced oxidative stress is highly prevalent. Such energy deficits can exacerbate pain sensitivity, and therapeutic strategies targeting mitochondrial function may offer new avenues protecting affected individuals against the pain chronification [67].

Physical exercise plays a pivotal role in mitigating mitochondrial dysfunction through pre-habilitation strategies. These interventions, which involve personalised exercise regimens tailored to individual metabolic profiles, aim to boost mitochondrial biogenesis and improve bioenergetic capacity. Exercise enhances reserve capacity in mitochondria, which is crucial in preventing energy crises during physical stress, helping to manage chronic diseases effectively [68]. Moreover, pre-habilitation strategies emphasise the importance of mitochondrial adaptations in response to exercise, particularly in patients with metabolic or mitochondrial pathologies [40].

## When and how the condition after BC treatments can be defined as suboptimal health?

Suboptimal health (SH) can be easily defined as the reversible phase of the health to-disease transition. Consequently, the time-frame between the SH condition onset and related clinically manifested disorder is the operational window to protect the affected person against the disease [69].

## Proposed mitochondria-based holistic 3PM approach

In the context of mitochondrial health and its relevance to predictive, preventive, and personalised medicine (PPPM or 3PM), the following sections explore various facets related to patient profiling, predictive measurements, targeted prevention, and treatments, as well as the importance of psychological supervision, the telephone consultations, and improving 3PM literacy.

### Phenotyping and individualised patient profiles

Phenotyping is central to creating an individualised patient profile that considers genetic, phenotypic, and lifestyle factors influencing mitochondrial health. Mitochondria can show varied responses to stressors such as oxidative damage or inflammation depending on individual susceptibility. Personalised profiles allow identifying patients who are more susceptible to deviation from the balanced functioning of mitochondria to suboptimal mitochondrial function. There are several approaches to determine the personalised profile. The most common ones are questionnaire and assessment the Mitochondrial Health Index (MHI). These profiles provide insights into mitochondrial capacity and bio-energetic efficiency, allowing adapted strategies for targeted prevention and treatments tailored to the person [40, 70].

### Predictive measurements

Predictive diagnostics, which utilise biomarkers such as cf-mtDNA and/or bio-energetic efficiency, aim at identifying patients at-risk and patients’ stratification in primary (protection against health-to-disease transition) and secondary (protection against disease progression). This allows for cost-effective early interventions, namely, targeted prevention and treatments tailored to individualised patient profiles.

### Targeted prevention

Targeted prevention, aligned with 3PM principles, is designed to mitigate the onset of diseases by addressing mitochondrial dysfunction early. For high-risk populations of breast cancer survivors, preventive strategies could include lifestyle adjustments (e.g., diet, exercise) alongside the use of nutraceuticals such as CoQ10, resveratrol, or alpha-lipoic acid, which are known to protect mitochondrial function and enhance bioenergetics. These interventions have to be tailored to individualised patient profiles utilising specialised questionnaire and well measurable indicators of mitochondrial health, homeostasis, stress, and burn-out.

### Rehabilitation algorithms tailored to individualised patient profiles

To be effective, treatments focused on mitochondrial health have to be tailored to individualised patient profile. In order to accurately design individualised rehabilitation protocols for post-breast cancer patients who underwent oncological treatments known to impact mitochondrial function and health, below presented approach is recommended.

#### Diagnostic approach based on the assessed mitophagy levels

Measuring systemic mitophagy levels is a reliable indicator of mitochondrial health and physiologic homeostasis. Contextually, cf-mtDNA profiles reflect individualised reaction towards imbalanced stress overload, mitochondrial stress, efficacy of compensatory mechanisms, and/or mitochondrial damage. Too high cf-mtDNA levels may indicate compensatory regulation of mitochondrial homeostasis under stress conditions, while too low levels may reflect severe mitochondrial decline and mitochondrial burn-out [36].

cf-mtDNA is detectable in blood; however, tear fluid analysis is emerging as a reliable non-invasive alternative to blood for diagnostics of mitochondrial health and homeostasis [36, 38]. Tear fluid collection is offering greater stability in molecular patterns compared to blood, making it a suitable medium for long-term monitoring of mitochondrial health, with annual or more frequent assessments to track changes in mitochondrial function over time [36]. By analysing cf-mtDNA levels in the tear fluid, mitochondrial damage and impairments can be detected early at the stage of reversible damage to health, which is crucial for tailoring preventive measures and individualised treatments [36, 66, 71, 72]. According to experience collected by the leading experts in the area, namely, Golubnitschaja et al. [36, 38, 39, 71], an accuracy of individualised data interpretation depends directly on the quality of corresponding data bank.

Patients undergoing tear fluid analysis and interpretation of the cf-mtDNA can be roughly sub-grouped as follows:

- 1st group within the reference range.
- 2nd group above optimum demonstrating elevated mitophagy levels.
- 3rd group below optimum demonstrating decreased mitophagy levels.

#### Recommendations provided for stratified patient groups

##### 1st group within the reference range

Patients with mitophagy levels within the normal range demonstrate balanced mitochondrial function. The goal here is to maintain mitochondrial health and to support mitochondrial bioenergetics.

Generalised recommendations for natural supplementary substances are [38]:

- Vitamin B complex with protective properties towards mitochondrial enzymatic function.
- Omega-3 Fatty Acids: Support mitochondrial membrane integrity with significant anti-inflammatory potential.

##### 2nd group with abnormally elevated mitophagy levels

Abnormally elevated cf-mtDNA in tear fluid frequently indicates compensatory mitochondrial over-activity and mitochondrial stress usually accompanied by increased production of reactive oxygen species (ROS) and oxidative damage to sub/cellular components. The goal here is to mitigate mitochondrial stress and to stabilise physiologic mitochondrial homeostasis.

Generalised recommendations for natural supplementary substances are [38]:

- Coenzyme Q10 (CoQ10): Supports the electron transport chain, enhancing ATP production while reducing ROS production.
- Alpha-lipoic acid: Provides antioxidant protection to reduce mitochondrial stress.
- N-acetylcysteine (NAC): Aids in glutathione synthesis, detoxifying ROS and protecting mitochondrial integrity.
- Resveratrol: Promotes mitochondrial biogenesis and reduces ROS.

##### 3rd group with abnormally lowed mitophagy levels

Abnormally low cf-mtDNA levels in the tear fluid may indicate mitochondrial burn-out associated with impaired energy production and highly increased cellular stress. The aim is to restore mitochondrial health and physiologic homeostasis by promoting mitophagy and to compensate cellular energy demands associated with mitochondrial burn-out.

Generalised recommendations for natural supplementary substances are [38]:

- Fisetin: Enhances mitophagy, clearing dysfunctional mitochondria.
- Quercetin: Promotes mitophagy and protects against oxidative stress.
- Creatine: Provides an alternative energy source to support cells with impaired mitochondrial function.
- PQQ (Pyrroloquinoline Quinone): Enhances mitochondrial biogenesis, maintaining an optimal mitochondrial population.
- L-carnitine: Facilitates the transport of fatty acids into mitochondria for energy production.

Despite generalised recommendations provided above, it is important to keep in mind that individualised patient coaching is crucial utilising detailed patients’ interview, accurate data interpretation, and follow-up consultations [71].

### Long-term monitoring

The use of tear fluid for analysing mitochondrial homeostasis offers a big advantage for long-term monitoring. Patients can undergo regular (several times a year) assessments to track changes in their whole body mitochondrial health status. This allows for timely adjustments in treatment protocols, improving personalised health management over time. This protocol supports a personalised approach to optimising mitochondrial health, aligning with the principles of predictive, preventive, and personalised medicine (PPPM) [40].

### Who can benefit from cf-mtDNA monitoring and mitochondrial health intervention?

Mitochondrial health intervention based on the optimalisation of mitochondrial function through dietary supplements can contribute to improving mitochondrial health and reducing fatigue. Supplements like CoQ10, resveratrol, and alpha-lipoic acid have demonstrated potential in supporting energy metabolism and reducing oxidative stress, both of which are essential for maintaining cellular vitality [38]. However, caution is needed in breast cancer survivors with a high probability of disease recurrence. In such cases, the use of mitochondrial-targeting supplements may inadvertently enhance the survival capacity of potential residual cancer cells. This is because mitochondria are essential for both healthy and tumour cells. Therefore, any intervention aimed at influencing systemic (whole body) mitochondrial function must be carefully assessed avoiding any support of the residual cancer cells proliferation and tumour recurrence, specifically in patients with an increased risk of the tumour recurrence such as those with high lymph node burden, large tumour size, and oestrogen receptor-positive tumours [73]. Since the primary approach in medicine is to avoid procedures which may be potentially harmful for the patient, optimising mitochondrial health with the aim of addressing suboptimal health can only be recommended for breast cancer survivors who have a long-term disease-free prognosis. For these patients, evident benefits by improving mitochondrial health resulting in stabilised health conditions and significantly improved quality of life are of high priority.

### Psychological supervision

Psychological health is closely linked to mitochondrial function, with stress and mental health disorders often exacerbating mitochondrial dysfunction. Psychological supervision, therefore, becomes a key element in maintaining mitochondrial health, especially in patients recovering from conditions like ischemic stroke, where stress and cognitive impairments can adversely affect recovery outcomes. Psychological support, when integrated into a 3PM strategy, not only improves patient mental well-being but also contributes to better overall mitochondrial health, reducing stress-induced damage.

### Telephone consultation and surveys

In the framework of personalised medicine addressed to breast cancer survivors, telephone consultations and surveys serve as vital tools for patient monitoring and follow-up. They allow for continuous assessment of health status outside the clinical setting, enabling early detection of changes in patient health and timely intervention. Surveys can help assess lifestyle factors, adherence to therapeutic regimes, and mental health status, while consultations provide an avenue for ongoing psychological support and medical advice.

### 3PM literacy

Promoting 3PM literacy amongst oncologists and other healthcare professionals involved in breast cancer survivors’ follow-up is essential for the successful implementation of individualised treatments targeting mitochondrial health. This literacy ensures that both professionals and patients understand the value of predictive diagnostics, personalised treatments and the role of lifestyle factors in maintaining mitochondrial function. Improved education on 3PM principles fosters better patient engagement and adherence to preventive strategies, contributing to a long-term success of personalised healthcare.

### Generalised innovative rehabilitation concepts for the mitochondria-based 3PM approach

**Fig. 2:**
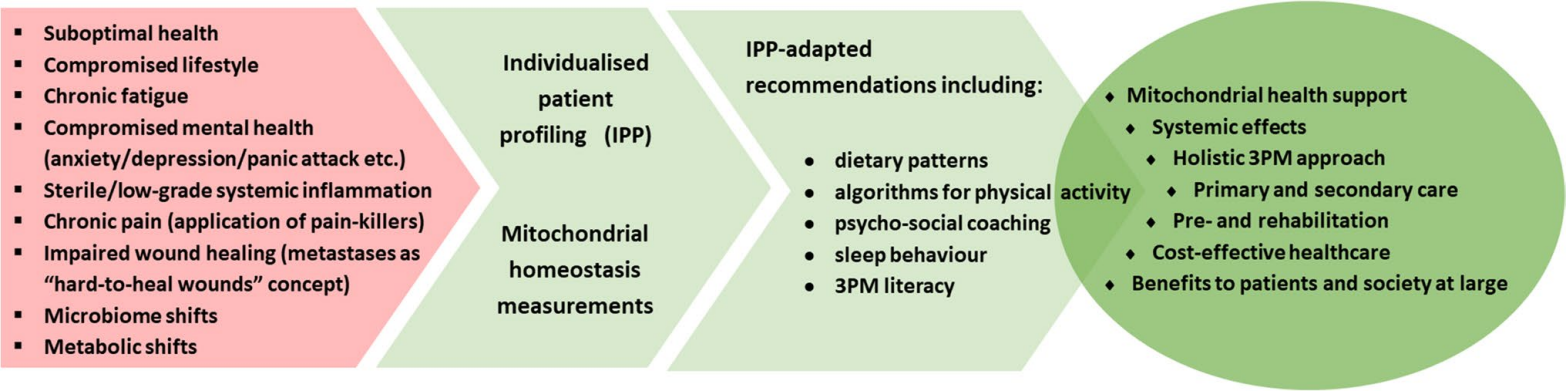
Mitochondria-based holistic 3PM approach is proposed for innovative cost-effective pre- and rehabilitation concepts

Innovative pre- and rehabilitation concepts are summarised in Fig. 2. Symptoms in common are summarised for treated cancer-survivors as well as for patient cohorts suffering from systemic disorders such as CVDs, neurodegenerative and metabolic ones, amongst others, which mitochondrial health and homeostasis are highly relevant for. Mitochondrial homeostasis is considered the key parameter for objective measurements of the patient’s health status (optimal *versus* suboptimal one), therapy success monitoring, coaching of adapted physical activities, and cost-effective pre- and rehabilitation performance. These evidence-based concepts utilise previously published field-relevant research data analysis and interpretation [68].

## Data Availability

No datasets were generated or analysed during the current study.
